# Supplementary material for: Prevalence and risk factors for multi-drug resistant Escherichia coli among poultry workers in the Federal Capital Territory, Abuja, Nigeria
Source: PLoS One. 2019 Nov 21;14(11):e0225379. doi: 10.1371/journal.pone.0225379 (PMC6872178; doi:10.1371/journal.pone.0225379)
Supplement: S1 Appendix — Survey of Risk factors for Multi-drug resistant E. coli in Poultry and Poultry workers in FCT, North Central Nigeria. (PDF) [file pone.0225379.s001.pdf]

APPENDIX I

Survey of Risk Factors for Multi-drug Resistant *E. coli* in Poultry and Poultry Workers in FCT, North Central Nigeria

INTERVIEWER-ADMINISTERED QUESTIONNAIRE

Introduction and consent section:

INFORMED CONSENT

Greetings. My name is Dr. Mabel Aworh-Ajumobi and I am a post graduate student of the Department of Veterinary Public Health and Preventive Medicine, Ahmadu Bello University, Zaria.

My team is conducting a study that asks questions about working with chickens in the poultry farm or live bird market (LBM). We would also be collecting a very little quantity of your stool sample which will be tested for the presence of *E. coli* organism. This bacteria is sometimes present in chickens which are slaughtered at the farm or LBM and in the poultry farm or market environment. This bacteria can infect the Poultry farmer or Seller making him sick.

You will be provided a sample bottle to collect a little quantity of your stool sample which we will take to the laboratory for analysis. After the lab results are out, you will be told the result of this test. This study will take place between November, 2018 and April 2019. We would very much appreciate your participation in this study. This information will help the government to plan better health care services for the people of Nigeria. Whatever information you provide will be kept strictly confidential. Participation in this study is voluntary, and if we should come to any question you don't want to answer, just let us know and we will go on to the next question. However, we hope that you will participate in this study since your views are important.

Would you like to participate in this study? **Yes** ☐ **No** ☐

Signature of respondent: ________________________

Date:________________

INTRODUCTION

The purpose of this questionnaire is to collect information that may assist in elucidating the risk factors that exposes poultry workers to *E. coli* infections in FCT, North Central, Nigeria.

DATA ON POULTRY FARM/LIVE BIRD MARKET

1. General Farm Data

**1.1**	Date of Survey (dd/mm/yy)	
**1.2**	Name of Investigator	
**1.3**	Name of Farm/LBM	
**1.4**	Age of Farm/LBM	
**1.5**	Name of Area Council	
**1.6**	Phone number of Farmer/Seller	
**1.7**	Location (GIS Coordinates)	

2. Farmer/Farm Manager/ Poultry Seller/ Poultry Processor Characteristics

**2.1**	Age	☐ 20 - 30 years ☐ 31 – 40 years ☐ 41 – 50 years ☐ Above 50
**2.2**	Gender	☐ Male ☐ Female
**2.3**	Marital Status	☐ Married ☐ Single
**2.4**	Education	☐ Informal/Adult Education ☐ Quranic ☐ Primary ☐ Secondary ☐ Tertiary
**2.5**	Primary profession	☐ Poultry farmer ☐ Civil servant ☐ Poultry seller ☐ Poultry processor ☐ Private sector ☐ Others Please specify ………………………
**2.6**	Membership of poultry association	☐ Yes ☐ No

**2.7**	Contact with animal health worker/veterinarian	☐ Yes ☐ No
**2.8**	**If Yes**	☐ Once a week ☐ Twice a month ☐ others pls specify ……….
**2.9**	How long have you been a poultry farmer/ processor/seller	☐ 1. Less than 1 year ☐ 2. Up to 5 years ☐ 3. Up to 10 years ☐ 4. More than 10 years
**2.10**	How often do you clear the poultry manure?	☐ 1. Every day ☐ 2. Twice a week ☐ 3. Once a week ☐ 4. Monthly
**2.11**	How do you dispose poultry manure?	☐ 1. In a pit ☐ 2. Bag and sell<br>3. Others specify ………………………..
**2.12**	Do you wash your hands with soap after contact with poultry and their faeces?	☐ 1. Yes ☐ 2. No ☐ 3. Sometimes ☐ 4. Never
**2.13**	Do you wash your hands with soap after using the toilet?	☐ 1. Yes ☐ 2. No ☐ 3. Sometimes ☐ 4. Never
**2.14**	What is your source of drinking water	☐ 1. Borehole ☐ 2. Well ☐ 3. River ☐ 4.Tap☐ 5. Others specify ………………………..
**2.15**	Do you boil your water before drinking?	☐ 1. Yes ☐ 2. No
**2.16**	Have you had diarrhoea in the last 3 months?	☐ 1. Yes ☐ 2. No
**2.17**	If yes, how often?	☐ 1. Frequently ☐ 2. Sometimes
**2.18**	Do you have any knowledge of diarrhoea illnesses?	☐ 1. Yes ☐ 2. No
**2.19**	If yes, name them	
**2.20**	Do you know of some diseases that can be transmitted from animals to human?	☐ 1. Yes ☐ 2. No
**2.21**	If yes, name them	

3. Awareness on Multi-drug Resistant *E. coli* / Colibacillosis

**3.1**	Are you aware of *E. coli* infection in poultry farms or affecting chickens?	☐ 1. Yes ☐ 2. No

**3.2**	Have you ever had *E. coli* infection on your farm/ in your chickens?	☐ 1. Yes ☐ 2. No
**3.3**	**If yes, what were the signs/ symptoms?**	☐ 1. Chick mortality ☐ 2. Diarrhoea<br>☐ 3. Reduction in Egg production<br>4. Others specify ………………………..

4. **Sample Stock Details**

**4.1**	**Flock size**	
**4.2**	**Breed of flock**	
**4.3**	**Age of flock (weeks)**	
**4.4**	**Source of day-old chicks**	
**4.5**	**System of Production**	☐ 1. Deep litter ☐ 2. Battery cage
**4.6**	**If Deep Litter, Litter type**	☐ 1. Wood shaving<br>☐ 2. Saw dust<br>☐ 3. Other, specify……………………………………..
**4.7**	**Source of Feed**	☐ 1. Locally compounded<br>☐ 2. Commercial
**4.8**	**Source of drinking water**	☐ 1. Pipe-borne ☐ 2. Well ☐ 3. Bore hole ☐ 4. Others specify ………………………………..
**4.9**	**Do you add medication to feed**	☐ 1. Yes ☐ 2. No<br>If yes list the medications…………………………………………………..
**5.0**	**Do you add medication to water**	☐ 1. Yes ☐ 2. No<br>If yes list the medications…………………………………………………..

5. **Disease Management**

**5.1**	Do you vaccinate your birds?	☐ 1. Yes ☐ 2. No

**5.2**	Has there been any disease outbreak on your farm or LBM?	☐ 1. Yes ☐ 2. No
**5.3**	Was there any treatment provided for the outbreak?	☐ 1. Yes ☐ 2. No
**5.4**	If yes name medications	
**5.6**	Any antibiotics treatment in the last 2 weeks?	☐ 1. Yes ☐ 2. No
**5.7**	If yes, name antibiotics used	
**5.8**	How do you treat sick birds?	☐ 1. Call a Vet ☐ 2. Treat by yourself<br>☐ 3. Ask other farmers or poultry sellers on what to do<br>Others pls specify………………………………………………..
**5.9**	Management of dead birds	☐ 1. Bury on farm ☐ 2. Sell the birds ☐ 3. Throw over the fence ☐ 4. Throw in the pit ☐ 5. Incinerate

6. Farm Biosecurity/ LBM Biosecurity

**6.1**	Farm is fenced	☐ 1. Yes ☐ 2. No
**6.2**	Floor type	☐ 1. Concrete ☐ 2. Others pls specify………..
**6.3**	Poultry waste management	☐ 1. On farm /In Market ☐ 2. Off farm/market
**6.4**	Poultry by-product disposal	☐ 1. On farm/In market ☐ 2. Off farm/market
**6.5**	Rodent Control (Observation/bait)	☐ 1. Yes ☐ 2. No
**6.6**	Presence of other animals on farm/near LBM	☐ 1. Yes ☐ 2. No
**6.7**	Presence of other poultry farms within 1km radius of farm or LBM	☐ 1. Yes ☐ 2. No
**6.8**	Presence of wild birds on the farm/LBM	☐ 1. Yes ☐ 2. No
**6.9**	Are there foot dips provided at entrance of houses?	☐ 1. Yes ☐ 2. No

**6.10**	How frequently are the disinfectants used in the dip changed?	☐ 1. Every week ☐ 2. Every month ☐ 3. Other, Specify ……………………………………..
**6.11**	Do the doors, windows and other openings of the houses have wire mesh?	☐ 1. Doors only ☐ 2. Windows only ☐ 3. Drain pipes only ☐ 4. All openings ☐ 5. No opening is wire meshed
**6.12**	Is there a changing room on the farm/market	☐ 1. Yes ☐ 2. No
**6.13**	Is there a lavatory on the farm/market?	☐ 1. Yes ☐ 2. No
**6.14**	Do staff of the farm work on other poultry farms	☐ 1. Yes ☐ 2. No
**6.15**	Types of cages used in LBM	☐ 1. Plastic crates ☐ 2. Wooden cages ☐ 3. Metal cages ☐ 4. Rafia cages

Thank you for your time.
