# Supplementary material for: Prevalence and risk factors for multi-drug resistant Escherichia coli among poultry workers in the Federal Capital Territory, Abuja, Nigeria
Source: PLoS One. 2019 Nov 21;14(11):e0225379. doi: 10.1371/journal.pone.0225379 (PMC6872178; doi:10.1371/journal.pone.0225379)
Supplement: S3 Appendix — (PDF) [file pone.0225379.s003.pdf]

Laboratory Protocol

Prevalence and Risk Factors for Multi-Drug Resistant *Escherichia coli* among Poultry Workers in the Federal Capital Territory, Nigeria

Bacteriological Analysis

Isolation and identification of *E. coli* isolates

Enrichment of the samples

Briefly about one gram of human stool sample was inoculated in enrichment broth (buffered peptone water) and incubated at 37°C for 24 h. Thereafter, a loop-full culture from enrichment broth was streaked onto MacConkey's lactose agar and incubated at 37°C for 24 h. Suspected *E. coli* colonies, usually pink to red were picked and further streaked on Eosin Methylene Blue (EMB) agar (10).

Plating

Three well-isolated suspected *E. coli* colonies usually pink in colour were selected at random from the MacConkey lactose agar plate and then sub-cultured on to Eosin Methylene Blue (EMB) agar, with a sterile inoculating loop and incubated at 37°C under aerobic conditions for 24h. Colonies that are raised, moist with greenish metallic sheen, suggestive of *E. coli,* were then sub-cultured onto trypticase soy agar plates and incubated for 24h at 37°C under aerobic conditions for the isolation of pure cultures. All *E. coli* isolates were further screened using conventional biochemical tests (Triple sugar iron, Urease, Sulphur Indole motility, Methyl red, Voges–Proskauer, and Citrate utilization biochemical tests) (10). Isolates tentatively shown to be *E. coli* in the conventional

biochemical screening tests were subjected to further tests using commercially available biochemical test strip, Microbact GNB 24E (Oxoid, UK), for confirmation. This kit was used according to the Manufacturer's instructions.

Antimicrobial susceptibility profiling

The antibiotic susceptibility patterns of *E. coli* isolates were tested using the Kirby Bauer disk diffusion method and according to the Clinical and Laboratory Standards Institute guidelines (CLSI, 2018). Briefly, one colony of the test isolates from overnight cultures grown on trypticase soy agar was picked using sterile Pasteur loop and emulsified in sterile normal saline. The turbidity of the suspension was adjusted to 0.5 MacFarland's standard and then using sterile cotton swab, the bacteria was spread on Mueller Hinton agar to obtain a lawn culture. After air drying, commercially available antibiotic discs (Oxoid, UK) were placed 30mm apart and 10mm away from the edge of the plate and incubated at 37°C for 24hours. Inhibition zone diameter was measured, recorded and values interpreted using standard recommendations of the Clinical and Laboratory Standards Institute (CLSI, 2018). The isolates were tested using a panel of 16 antibiotics of different classes commonly used to treat human and animal bacterial infections namely Ampicillin (10µg), Amoxycillin/ clavulanic acid (20/10µg), Tetracycline (30µg), Gentamicin (10µg), Cefuroxime (30µg), Streptomycin (10µg), Chloramphenicol (30µg), Nalidixic acid (30 µg), Sulfamethoxazole-trimethoprim (10µg), Cephalothin (30µg), Nitrofurantoin (300µg), ceftriaxone (30µg), imipenem (10µg), colistin (10µg), ceftazidime (30µg) and Cefotaxime (30µg). A standard reference strain of *E. coli* (ATCC 25922), sensitive to all antimicrobial drugs being tested, would be used as a control. We defined multi-drug

resistance (MDR) as resistance to three or more classes of antibiotics used for treating poultry diseases.

Determination of Multiple Antibiotic Resistance (MAR) index

The Multiple antibiotic resistance (MAR) index for each isolate was determined using the formula MAR = a/b, where *'a'* is the number of antibiotics to which the test isolate was resistant to and *'b'* is the total number of antibiotics to which the test isolates were subjected (11).

Detection of ESBL phenotype by double disk methods

All *E. coli* isolates were screened for the production of Extended-Spectrum Beta-Lactamase (ESBLs) by using double disk method as described by (12). From the pure cultures of bacteria grown overnight on MacConkey agar, a suspension matching 0.5 McFarland standard (1.5 x 108 CFU/ml) was made in normal saline. Using sterile cotton swab, the bacteria was spread on Mueller Hinton agar to obtain a lawn culture. After allowing the plate to dry, the antibiotic disk amoxycillin/clavulanic acid 20+10 mg was placed at the center of the plate. Next, one cefotaxime 30 mg disk and disk of ceftazidime 30 mg were placed 15mm apart from the edge of amoxycillin/ clavulanic acid disk. The plates are incubated in air at 37°C for 16-18 hours. Following growth, the diameter of the zones of inhibition around the disks were measured and recorded. Any *E. coli* isolate that showed an increase in the zone of inhibition around either ceftazidime or cefotaxime (i.e., > 5 mm towards the disc of amoxicillin-clavulanate) was interpreted as positive for the ESBL production. *E. coli* ATCC 25929 and *K. pneumoniae* ATCC 700603 were used as negative and positive controls, respectively.
