# Supplementary material for: Prevalence and risk factors for multi-drug resistant Escherichia coli among poultry workers in the Federal Capital Territory, Abuja, Nigeria
Source: PLoS One. 2019 Nov 21;14(11):e0225379. doi: 10.1371/journal.pone.0225379 (PMC6872178; doi:10.1371/journal.pone.0225379)
Supplement: Attachment — Submitted filename: Response to Reviewers.docx [file pone.0225379.s004.docx]

25 October 2019

**The Academic Editor,**

**PLOS ONE Journal.**

Dear Editor,

This is to acknowledge the effort of the editorial team for their immense contributions towards transforming the manuscript to its present form. All the observations raised and the corrections as well as comments made by the reviewers are appreciated and have been addressed.

Moreover, point-by-point response to reviewers’/editorial comments have been addressed as described in the appendix.

Should you require additional information or if you have any questions, please feel free to contact me. I will be pleased to hear from you.

Yours sincerely,

**Dr. Mabel K. Aworh** (Corresponding author)

mabelaworh@yahoo.com, maworh@ncsu.edu

+234 -803 237 7831, 919 741 1749

**Reviewer/Editorial Notes and Responses**

| **S/N** | **Comments** | **Authors’ response** |
| --- | --- | --- |
| 1. | **Journal Requirements**  Please ensure that your manuscript meets PLOS ONE's style requirements, including those for file naming. The PLOS ONE style templates can be found at: | Many thanks for your feedback. This has been addressed and the manuscript has been revised to ensure it meets PLOS ONE’s style requirements using the templates provided. |
| 2. | In your Methods section, please provide additional location information, including geographic coordinates for the data set if available | We appreciate these comments of the editorial staff. This has been addressed in the **Methods section** with the following statements.  ***This section now reads***:  This study was conducted in Abuja Municipal Area Council (9.0612° N, 7.4224° E) and Kuje Area Council (8° 52' 46.27" N, 7° 13' 39.22" E) with the largest poultry population of approximately 3.812,288 [18].  Kindly refer to page 6, lines 157 – 160. |
| 3. | Please include copies of the survey questions or questionnaires used in the study, in both the original language and English, as Supporting Information, or include a citation if they have been published previously. | This comment by the editorial staff is noted. Based on the editorial staff’s comment, a copy of the **questionnaire** has been provided as supplemental file.  Kindly refer to **Supporting Information section**, page 25, lines 631 – 632 |
| 4. | **Reviewer #1**: I think that this is a local issue not a global. | This is well received and has been addressed under **Introduction section.**  ***This section now reads***:  In developing countries including Nigeria, many families depend on poultry production as a means of income and livelihood due to the increased consumption of poultry products [5]. Antimicrobial drugs are also readily available over the counter without prescription because access to veterinary drugs is presently not being regulated in the country, thereby encouraging the use of these drugs by poultry farmers indiscriminately in production. Poultry farmers continue to use antibiotics in poultry feed or water for prophylaxis, treatment of diseases and as growth promoters in Nigeria [6,7]. Commonly used antibiotics in poultry production in Nigeria include; oxytetracycline, neomycin, enrofloxacin, doxycycline, gentamicin, colistin, streptomycin, tylosin, ciprofloxacin, nitrofurans and chloramphenicol. This is similar to the practice in most countries [7].  Kindly refer to page 4, lines 99 – 108. |
| 5. | English needs intensive revision. | This very important suggestion by the reviewer is appreciated. We have revised the manuscript accordingly. |
| 6. | Authors should clearly mention the study hypothesis in the introduction. | We quite appreciate the reviewers for their comments in this regard. The introduction section has now been modified to reflect the hypothesis for this study.  ***This section now reads***:  We hypothesized that poultry harboring drug resistant *E. coli* aids in the transmission of the pathogen to human workers who are exposed in farms and live bird markets.  Kindly refer to page 5, lines 145 – 146. |
| 7. | **Reviewer #2:** Page 4 Line 90: ESBL should be written in full | This is well received and has been addressed under **Introduction section**. Kindly refer to page 4, lines 111 - 112. |
| 8. | Page 4 Lines 91-92. Authors should spell out the socioeconomic and public health implications | This is well received and has been addressed under **Introduction section**.  ***This section now reads***:  Resistance to commonly used antibiotics has major socioeconomic and public health implications. The socioeconomic implications of AMR include increased cost and duration of treatment while the public health implications include decreased ability to treat common infections resulting in increased human suffering and ultimately death [9–11].  Kindly refer to page 4, lines 114 - 117. |
| 9. | Page 5 Line 116: Spell out FCT | This is well received and has been addressed under **Introduction section**. Kindly refer to page 6, line 153. |
| 10. | Page 5 Line 122: Census data from 2006 is too old. Needs an update | This is well received and has been addressed under **Methods section**.  ***This section now reads***:  Based on data obtained from the National Bureau of Statistics, the population projection for FCT for 2019 is 4,464,785 people [17].  Kindly refer to page 6, lines 156 - 157. |
| 11. | Page 6 Sampling: the total number of individuals sampled should be stated here | This is well received and has been addressed under **Methods section**.  Kindly refer to page 7, line 188. |
| 12. | Page 7 Line 168 Delete (1.0g) | This correction has been made. Kindly refer to page 8, line 210. |
| 13. | Consistency is needed in referencing Oxiod UK throughout the text | This correction has been made.  Kindly refer to page 9, line 238. |
| 14. | Authors should write in full the first time before using acronyms. A problem throughout the text: Page 8: Line 190 CLSI | This is well received and has been addressed under **Methods section**, page 9, line 240 -241. |
| 15. | Statistical Analysis: Authors should remove the female record and reanalyze the data. | This is well received and has been addressed under **Results section**  ***This section now reads***:  Of the 122 poultry workers we sampled, 99.2% (n=121) were male. The analysis presented in the results section was based on 121 males whose ages ranged from 18 to 67 years. The mean age of study participants was 30.6 ± 9.7years. The majority of workers were married: 54.6% (n=66), had secondary education: 57.9% (n=70) and worked on a poultry farm: 62.0% (n=75) (**Table 1**).  **Prevalence of Multidrug Resistant *E. coli* among poultry workers**  A total of 48 (39.7%) individuals tested positive for MDR *E. coli*. A majority of these, 62.5% (n=30) resided in Abuja Municipal Area Council (**Fig 1**). The highest prevalence was among farm-workers (POR = 2.7, 95% Confidence Interval [CI] =1.3 – 5.7; p = 0.01) when compared to poultry-sellers.  Kindly refer to pages 10 and 11, lines 282 - 294. |
| 16. | Authors should address how antibiotics are used in poultry in Nigeria: As prophylaxis, treatment of diseases or growth promotion? | This is well received and has been addressed under **Introduction section**  ***This section now reads***:  Poultry farmers continue to use antibiotics in poultry feed or water for prophylaxis, treatment of diseases and as growth promoters in Nigeria [6,7]. Commonly used antibiotics in poultry production in Nigeria include; oxytetracycline, neomycin, enrofloxacin, doxycycline, gentamicin, colistin, streptomycin, tylosin, ciprofloxacin, nitrofurans and chloramphenicol. This is similar to the practice in most countries [7].  In 2018, Nigeria’s National Agency for Food and Drugs Administration and Control (NAFDAC) issued a ban on the use of antibiotics as growth promoters in animal feeds.  Kindly refer to pages 4, lines 104 - 110. |
| 17. | What antibiotics are included in the feed if any? | This is well received and has been addressed under **Introduction section**  ***This section now reads***:  Commonly used antibiotics in poultry production in Nigeria include; oxytetracycline, neomycin, enrofloxacin, doxycycline, gentamicin, colistin, streptomycin, tylosin, ciprofloxacin, nitrofurans and chloramphenicol. This is similar to the practice in most countries [7].  Kindly refer to page 4, lines 105 - 108. |
| 18. | Conflict of interest from the authors were not declared Statement of availability of data was not made | This is well received and has been addressed.  ***These sections now read***:  **Competing interests**  The authors have declared that there are no competing interests.  **Data Availability Statement**  All relevant data are within the paper and also available as supporting information (S2 Appendix).  Kindly refer to, page 20, lines 492 – 493 (Competing Interests)  and lines 495 – 497 (Statement of Availability of Data) |
